# Ameliorative Effects of Anti-Clostridial Egg Yolk Antibodies (*IgYs*) in Experimentally-Induced Avian Necrotic Enteritis

**DOI:** 10.3390/ani12101307

**Published:** 2022-05-20

**Authors:** Zain Ul Abadeen, Muhammad Tariq Javed, Tariq Jamil, Azam Ali Nasir

**Affiliations:** 1 Department of Pathology, Faculty of Veterinary Science, University of Agriculture, Faisalabad 38040, Pakistan; mtjaved@uaf.edu.pk; 2 Institute of Bacterial Infections and Zoonoses (IBIZ), Friedrich-Loeffler-Institut (FLI), 07743 Jena, Germany; tariq.jamil@fli.de; 3 Anaerobe Section, Veterinary Research Institute (VRI), Lahore Cantt 54810, Pakistan; azamalinasir@gmail.com

**Keywords:** necrotic enteritis, egg yolk antibodies, *Clostridium perfringens*, microscopic, passive immunization, pathological

## Abstract

**Simple Summary:**

Necrotic enteritis (NE) is an important enteric infection in poultry. Since the ban on the use of antimicrobials growth promoters, NE has re-emerged significantly in commercial poultry. The current study aimed to evaluate the ameliorative effects of anti-clostridial egg yolk antibodies (EYAs) in NE. Passively immunized birds showed improved behavioral signs and gross and microscopic lesions. Although EYAs showed ameliorative potential in our study, studies regarding safe production and reliable application remain overdue.

**Abstract:**

The present study was planned to evaluate the ameliorative effects of egg yolk antibodies (EYAs) in broiler chicken. For this purpose, 80-day-old broiler chickens were divided into four groups (A–D), where group A was kept as negative control. Experimental infection with *C. perfringens* (1 × 10^8^ cfu/mL) was induced via oral route on days 17, 18 and 19 of the experiment in groups B, C and D. Groups C and D were passively immunized by anti-clostridial *IgYs* @ 1 mL per bird via oral and oral and intramuscular (I/M) routes respectively, on days 21 to 24, and on days 22 and 24 of the experiment, respectively. Two necropsies were performed (the first on day 26th and the second on day 35th). Birds in group B showed behavioral signs e.g., laziness, depression and diarrhea, gross post-mortem lesions e.g., increase in the relative weights (RW), due to acute swelling and congestion of liver and kidneys and ballooning and hemorrhages of jejunum and microscopic lesions e.g., congestion and necrosis in liver and kidneys’ parenchyma and disrupted epithelium with fewer goblet cells in jejunum, compared to the group A. Birds in groups C and D, showed significant improvements in clinical and behavioral signs, RW of liver, kidneys and jejunum, swelling, congestion and mononuclear cells’ infiltration in liver and kidneys and damages in the jejunal-wall, compared to group B. The most significant changes were found in birds of group C. Our study revealed ameliorative effects of EYAs on certain biological parameters however, further studies would be needed to justify a safer production and a reliable application of EYAs in NE outbreaks.

## 1. Introduction

Over the years, the Pakistani poultry industry has emerged to be one of the most successful national industries, with an average annual growth rate of 7.5% since 2012 [1]. Infectious disease outbreaks of various bacterial, viral and fungal origin, e.g., necrotic enteritis (NE), avian influenza (AI) and aspergillosis have posed a significant economical and health threat to this industry. NE, caused by *Clostridium (C.) perfringens*, is an important bacterial disease of the gastrointestinal (GI) tract in birds that may cost up to 5–6 billion USD per annum the global poultry industry [2]. This bacterium is a Gram-positive anaerobic bacillus, which resides as normal gut microbiota [3]. *C. perfringens* is grouped into five toxinotypes (A–E) depending upon the type of toxins produced e.g., alpha, beta, epsilon, iota, and enterotoxin [4]. Recently, another type “G” was described in birds producing both alpha and a pore-forming toxin known as NetB [5]. However, *C. perfringens* type A negative for *netB*, has been isolated from NE infected birds [6]. NE shows signs of dullness, diarrhea and poor body condition with gross post-mortem lesions, i.e., ballooned and hemorrhagic intestines and microscopic lesions, i.e., shortening of the villi with damaged epithelium in the intestines [7]. Antimicrobials targeting Gram positive bacteria are effective against acute NE in poultry.

Recently, NE has significantly re-emerged due to a gradual shift in husbandry practices towards being more environmentally friendly, e.g., ban of the use of anti-microbial growth promoters (AGPs) by the European Commission (EC) of the European Union (EU) since 2006 and the human health-related antimicrobials in food animals by the United States Food and Drug Administration (FDA) since 2015 [8,9]. Hence, finding a safe alternative remains indispensable. Egg yolk antibodies (EYAs) or *IgYs* are termed as protective antibodies produced in the egg yolk of immunized hens that can be used to neutralize specific pathogens of enteric origin and help to improve growth performance traits in infected birds [10,11]. Currently, EYAs have potential application in various immunodiagnostic and therapeutic approaches used in animals and humans [12,13]. Keeping in view the protective role of EYAs, the current study aims to evaluate the influence of anti-clostridial EYAs on the gross and microscopic lesions found in infected broiler birds.

## 2. Materials and Methods

### 2.1. Production of Anti-Clostridial EYAs

Ten (10) white leghorn hens (40 weeks of age) were immunized by injecting 1mL of inactivated whole cell antigen (WCA) of *C. perfringens* in the breast muscle, as described previously in detail [14]. Booster doses were administered on 42nd and 44th weeks [15]. EYAs were extracted as water-soluble fraction (WSF) from the laid eggs and assayed by ELISA, as described previously with some modifications (coated antigen = *C. perfringens* whole cells (10 µg/mL); secondary antibody = Rabbit anti-chicken IgG conjugated with horseradish peroxidase (Sigma-Aldrich^®^, Burlington, MA, USA) [15,16,17]. The anti-clostridial EYAs were stored at −20 °C.

### 2.2. Experimental Design

For the experiment, eighty (N = 80) day-old broiler chickens (Ross-308, Aviagen, Newbridge, UK) were procured from a local hatchery and reared under standard management conditions at the Animal Care and Research Facility of the Department of Pathology, University of Agriculture (UAF), Faisalabad, Pakistan for 35 days. The birds were divided into four groups (A–D) of twenty birds each (n = 20) for conduction of the experiment (Table 1). Group A served as the negative control and was kept on a basal diet (crude protein = 21%) [18]. Group B acted as the positive (infection) control, whereas Groups C and D acted as the passively immunized groups with different routes of administration.

### 2.3. Experiental Infection

Feed included 250 g/kg of fish feed in groups B, C and D [19]. All groups except A received an experimental infection dose of *C. perfringens* type A @ 1 × 10^8^ colony-forming units (CFUs) on days 17, 18 and 19 of the experiment [20]. Groups C and D received anti-clostridial EYAs @ 1 mL/bird orally (on days 21 to 24) and I/M (on days 22 and 24 of the experiment), respectively (Table 1). Five birds from all groups were selected randomly each on days 26 and 35 of the experiment for necropsy.

### 2.4. Parameters under Study

#### 2.4.1. Clinical Signs and Behavioral Symptoms

A random scoring system (0–4) was developed to score the severity of clinical signs and behavior symptoms, e.g., the desire of feed and water, fecal consistency, and activeness of the birds, where 0 = minimum and 4 = maximum. For each week, a cumulative score was estimated to express the behavior changes at the end of the experiment.

#### 2.4.2. Mortality (%)

The death of any bird in the experimental groups was observed twice a day and recorded in a cumulative form of percentages (%) at the end of experiment.

#### 2.4.3. Relative Weight of Organ

Absolute and relative weights of liver, kidneys, and jejunum of the small intestine, from necropsied birds was recorded for comparison.

#### 2.4.4. Post-Mortem Examination

##### Gross Lesions

Gross lesions of jejunum were classified and scored as the absence of any lesion (−), intestine thin walled and friable (+), focal necrosis and small amount of gas accumulation (++), small patches of necrosis, blood flakes and gas accumulated (+++), sever necrosis of larger area, hemorrhages and more gas accumulation (+++), as described by Prescott 1979 [21]. Gross lesions of the liver, e.g., congestion (CN) and enlargement (EN) and kidneys, e.g., hemorrhages (HM) and mucosal damage (MD), were classified and scored as described by Jerzsele et al. 2012 with some modifications, i.e., absence of lesion (0%) = −, mild (≤10%) = +, moderate (10–20%) = ++, severe (20–30%) = +++, more severe (≥40%) = ++++ [22].

##### Histopathology

Histological examination was performed by fixing the tissue samples in 10% neutral buffered formalin (NBF) and washing, embedding, slicing and staining of the sections on a slide by hematoxylin and eosin (H & E) dyes [23].

### 2.5. Statistical Analysis

The data obtained were statistically analyzed by one-way analysis of variance (ANOVA) using the general linear model (GLM). A *p*-value of ≤0.05 was kept as the statistical significance level. The mean values were compared by Tukey’s test and for this purpose, SAS stat 15.1 (SAS Institute, Cary, NC, USA) was used [24].

## 3. Results

### 3.1. Clinical Signs and Behavioral Symptoms

During first and second weeks of the experiment, birds in all groups remained active and alert with signs of normal feed and water intake and fecal consistency. From the third week, birds in groups B, C and D showed signs of laziness and dullness, with watery droppings. From the fourth and fifth weeks, birds in group B showed signs of depression, lack of appetite and diarrhea. However, birds in groups C and D showed less severe signs compared to group B in this time (fourth and fifth weeks) (Table 2). Birds in group A remained active and showed signs of normal feed and water intake and fecal consistency throughout the experiment.

### 3.2. Mortality (%)

No mortality was found in birds of all groups during the first and second weeks of the experiment. Afterwards, group B showed the highest mortality (30%), followed by group D (20%) and group C (15%). No mortality was found in the group A birds throughout the experiment. The mortality rates were lower in passively immunized groups C and D when compared to group B, although the effect was not significant (*p* ≥ 0.05) among all groups (Table 3). The third week showed highest mortality (54%) rates in the experiment.

### 3.3. Relative Weight (RW) of Organs

During the postmortem examination on day 26, the carcass weight of birds showed a significant (*p* ≤ 0.05) variation among groups, with the lowest carcass weight in groups B, D and C, respectively, when compared to group A. Liver, kidneys and jejunum showed a significant (*p* ≤ 0.05) increase in the relative weight among groups, with the highest increase in group B, followed by groups D and C, when compared to group A (Table 4).

On day 35, the carcass weight of birds varied significantly (*p* ≤ 0.05) among groups, with the lowest values in group B, followed by groups D and C, when compared to group A. RW varied significantly (*p* ≤ 0.05) in liver among groups B and D; however, it did not vary significantly (*p* ≥ 0.05) among group C when compared to group A. In kidneys, it varied significantly (*p* ≤ 0.05) among group B only and did not vary significantly (*p* ≥ 0.05) among groups C and D when compared to group A. In jejunum, the RW varied significantly (*p* ≤ 0.05) among groups, with the highest values in group B, followed by groups D, C and A (Table 5).

### 3.4. Post-Mortem Examination

#### 3.4.1. Gross Lesions

**Figure 1:**
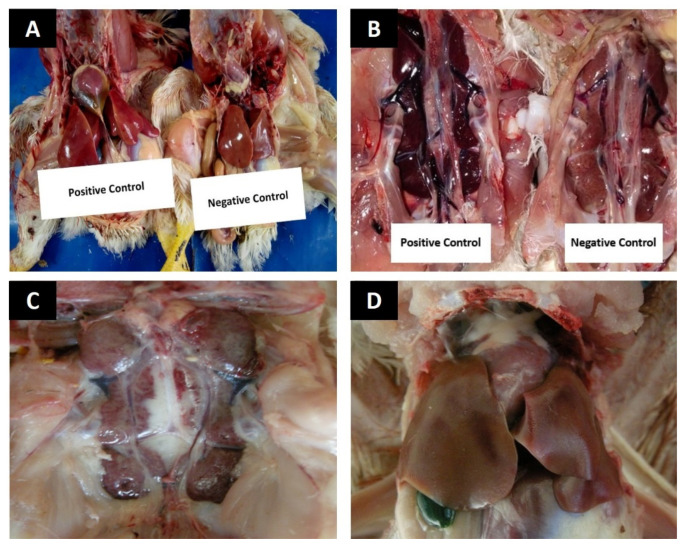
Photographs showing gross lesions in various organs (**A**) Swollen and congested liver from group B, while liver from group A has normal size and color (**B**) kidneys showing severe congestion and swelling from group B, while kidney has a normal appearance in group A (**C**) Kidney showing moderate swelling and congestion in group D (**D**) Liver showing mild congestion and swelling in group C.

**Figure 2:**
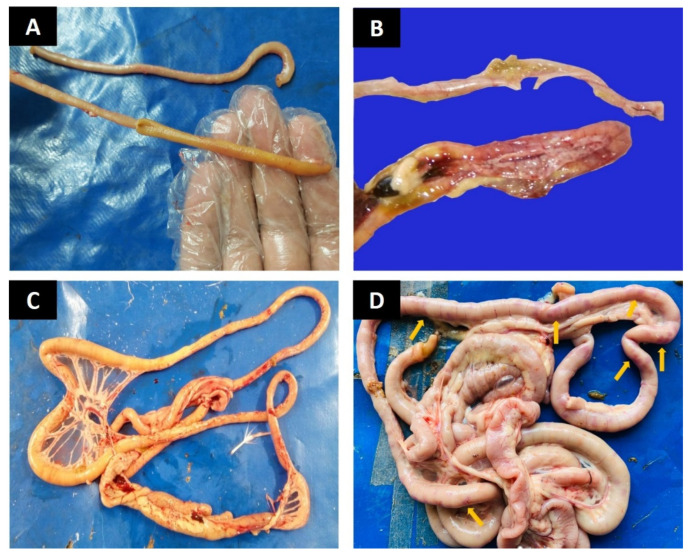
Photographs showing gross lesions of intestines (**A**) Normal appearance of intestinal mucosa and absence of any lesion in group A (**B**) Jejunum showing hemorrhages and perforations in the mucosa of group B (**C**) Moderate mucosal damage and congestion in intestine of group D (**D**) Jejunum showing areas of small hemorrhages visible from outside (arrows) in group C.

Liver, kidneys, and jejunum in group A birds appeared normal without showing any gross lesions (−) (Figure 1A,B and Figure 2A). In group B, the liver and kidneys showed acute swelling (+++) and congestion (Figure 1A,B). The jejunum appeared ballooned and severely hemorrhagic (+++) with thin-walled and damaged mucosa (Figure 2B). In group C, the liver and kidneys showed mild congestion (+) and moderate swelling (++) (Figure 1D). The jejunum showed moderate hemorrhages and moderate damages (++) to the mucosa (Figure 2D). In group D, the liver and kidneys showed moderate (++) swelling and congestion (Figure 1C) and moderate (++) mucosal damage with hemorrhages in jejunum (Figure 1C) (Table 6).

#### 3.4.2. Histopathology

##### Liver

**Figure 3:**
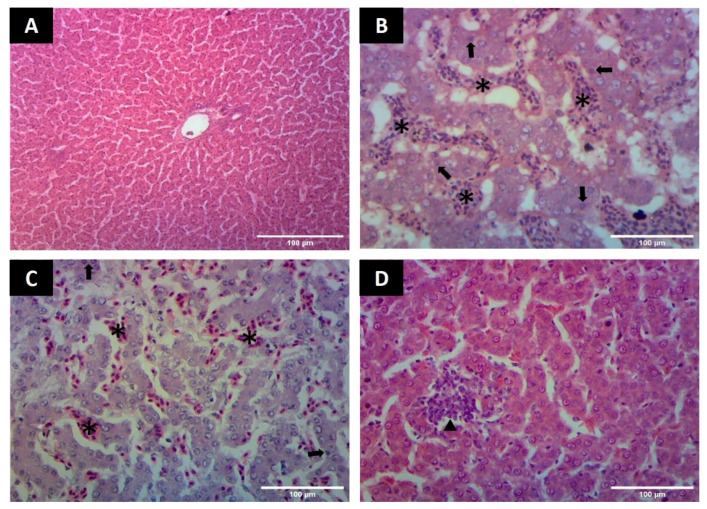
Photomicrographs of liver showing microscopic changes (**A**) Liver from group A showing normal appearance of hepatocytes arranged in hepatic cords (H & E staining 100×) (**B**) Liver in group B showing hepatic cell necrosis (arrows) and congestion indicated by the presence of RBCs (asterisk) in parenchyma (H&E staining 400×) (**C**) Liver in group B showing the area of congestion (asterisk) and disorientation of hepatic cords with hepatocytes necrosis (arrows) (H&E staining 400×) (**D**) Liver showing hepatocytes with prominent nuclei and presence of mononuclear cells (arrow heads) in group C (H&E staining 400×).

Birds in group A, showed normal hepatic parenchyma arranged in hepatic cords with normal oriented nuclei and prominent chromatin material (Figure 3A). Moderate to severe congestion was found in hepatic parenchyma in group B and hepatocytes swollen, disoriented causing sinusoidal spaces to appear less prominent. Moreover, hepatocytes contained pyknotic nuclei, indicating cell necrosis (Figure 3B,C). In groups C and D, mononuclear cells mildly infiltrated hepatic parenchyma, where hepatocytes appeared normal in morphology containing prominent nuclei (Figure 3D).

##### Kidneys

**Figure 4:**
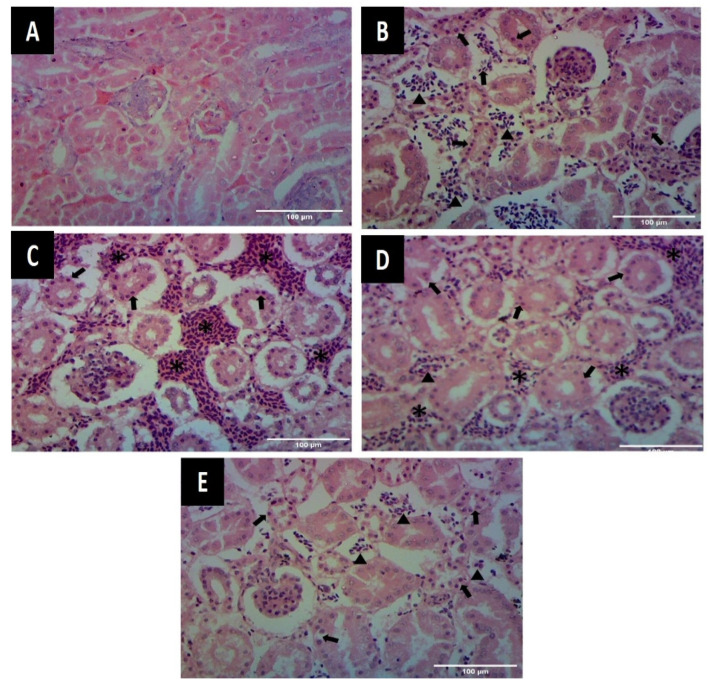
Photomicrographs of kidneys showing microscopic changes (**A**) Kidney from group A showing normal appearance of tubular epithelial cells and glomeruli (H&E staining 400×) (**B**) Kidney from group B showing presence of mononuclear cells and RBCs (arrow heads) and necrosis of tubular epithelial cells (arrows) (H&E staining 400×) (**C**) Kidney from group B showing areas of congestion (asterisk) and necrosis of tubular epithelial cells (arrows) (H&E staining 400×) (**D**) Kidney showing necrotic tubular epithelial cells (arrows), presence of mono-nuclear cells (arrow heads) and congested areas (asterisk) in group D (H&E staining 400×) (**E**) Kidney from group C showing the presence of few mononuclear cells (MC) and RBC (arrow heads) along with darkly stained nuclei of tubular epithelial cells at few places (arrows) (H&E staining 400×).

In group A, the renal parenchyma showed normal tubular epithelial cells with normally dilated and clear urinary spaces (Figure 4A). In group B, the tubular epithelial cells showed dark stained pyknotic nuclei, indicating cell necrosis and renal parenchyma with moderate to severe congestion, along with infiltration of mono-nuclear cells. Urinary spaces did not appear clear (Figure 4B,C). In groups C and D, renal parenchyma showed infiltration of mono-nuclear cells, dark stained nuclei in tubular epithelial cells and few areas of congestion, whereas the urinary spaces appeared clear and dilated (Figure 4D,E).

##### Jejunum

**Figure 5:**
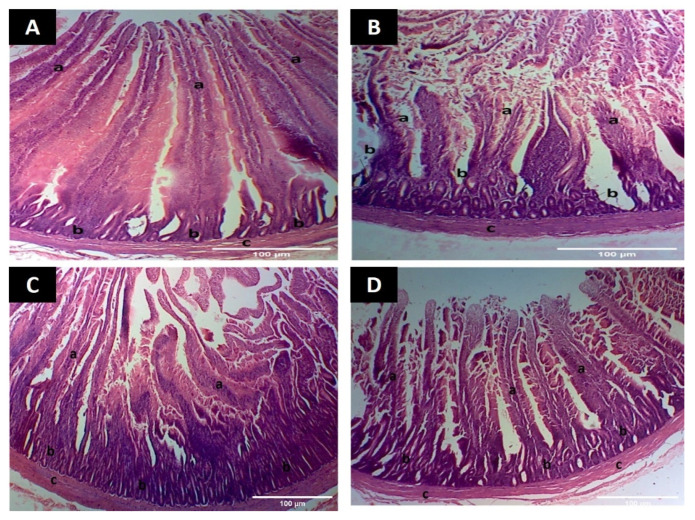
Photomicrographs of jejunum showing microscopic changes (**A**) Intestine from group A showing (a) developed villi having intact epithelium (b) goblet cells (c) submucosa (H&E staining 100×) (**B**) Intestine from group B showing (a) damaged villi with disrupted epithelium (b) fewer goblet cells (c) submucosa (H&E staining 100×) (**C**) Intestine from group C showing (a) developed villi (b) goblet cells (c) submucosa (H&E staining 100×) (**D**) Intestine from group D showing (a) villi with damaged epithelium at few places (b) goblet cells (c) submucosa (H&E staining 100×).

In group A, jejunal villi appeared normal with intact epithelium and goblet cells throughout the length (Figure 5A). In group B, jejunal epithelium appeared disrupted with damaged villi and few goblet cells (Figure 5B). In groups C and D, villi showed intact epithelium with fine development, goblet cells and normal submucosa (Figure 5C,D).

## 4. Discussion

Necrotic enteritis (NE), caused by *C. perfringens* type A, is an economically important infection in Pakistani poultry [6]. Previously, up to 25.37% prevalence was reported, highlighting the need of a safe and antimicrobial-free alternative to control and treat this infection in poultry. Keeping this necessity in mind, we tried to evaluate the beneficial effects of anti-clostridial EYAs therapy, as passive immunization has proved to be a potential alternative to antimicrobial therapy in various poultry-enteric infections [10]. Thus, anti-clostridial EYAs were administered in experimentally infected broiler birds via different routes and their potentially beneficial effects were studied, based on various clinical and histological parameters.

Birds in groups C and D significantly (*p* ≤ 0.05) improved clinical and behavioral signs including physical alertness, fecal consistency and appetite compared to group B, from the fourth and fifth weeks of the experiment, although there was not a significant (*p* ≥ 0.05) difference in the mortality pattern among all groups; birds in group B showed the highest mortality rate (30%) compared to groups C (15%) and D (20%). These changes in infected birds might be due to the toxins produced by *C. perfringens*, which could have directly influenced GIT, which might have indirectly affected fecal consistency, appetite and, hence, overall physical alertness [25,26]. Formerly, Ref [7]” investigated that oral administration with *C. perfringens* @ 4 × 10^8^ cfu (three times a day) for four consecutive days resulted in loss of body condition, reduced feed intake, diarrhea and depression in birds. Furthermore, Ref [27] reported emaciation, brown colored diarrhea, depression and reduced apatite in broilers after oral infection with 2 mL broth culture of *C. perfringens* @ 1.9 × 10^9^ cfu/mL for three alternate days. Similarly, oral gavage of *C. perfringens* administered in broilers resulted in ruffled feathers, movement reluctance, watery feces and depression [28,29]. All these paraments were important and observable indicators of birds’ health and well-being, which can be graded based on the evaluation system.

RW of the internal organs, i.e., liver, kidneys and jejunal parts of the small intestine, was significantly (*p* ≤ 0.05) higher in group B compared to group A, while significantly (*p* ≤ 0.05) lower in groups D and C compared to group B, but higher than group A, on the day 26 post-mortem examination. This meant the most damages in these organs occurred in group B and the birds in groups D and C showed significantly improved results but lesser than group A. The production of interleukin-1 during the inflammatory process might have resulted in anorexia and muscle wastage. The gut microflora is specifically targeted by the use of antibiotics to minimize the inflammatory process, but it is reported that EYA against specific neuropeptides help to stimulate the immune system to lower growth reduction [30,31].

The results of gross and microscopic studies in birds belonging to group B showed the presence of gross and histological alterations in various organs. Grossly, the liver and kidney were swollen and congested, while the intestines were gas-filled and had hemorrhagic mucosa with small areas of necrosis. The histological investigations of liver parenchyma showed the presence of mono-nuclear cells, cellular necrosis, diminished sinusoidal spaces and congestion. In renal parenchyma, the presence of condensed nuclei of tubular epithelial cells, congestion with dilated urinary spaces were observed. The presence of atrophied villi with damaged epithelium was observed in the intestine. The lumen of the intestines contained necrotic debris and submucosal congestion. These pathological alterations are probably due to the alpha (α) toxin produced by *C. perfringens*, which activates the arachidonic acid cascade to stimulate immune activity by producing various chemical mediators such as leukotrienes, thromboxane, and different activation factors for platelets aggregation and shrinkage of blood vessels. These clostridial toxins cause the degradation of intestinal mucosal membranes and in the liver produce tissue damage after reaching through hepato-portal circulation [26,32]. The results of present study agreed well with findings of [7], who reported that oral administration with *C. perfringens* (4 × 10^8^ cfu) produced gross lesions and histopathological changes in the intestines and liver of broilers. Furthermore, Ref [33] found severe gross and microscopic alterations in the liver, kidney and intestine of broiler birds after oral infection with 1 mL of broth culture of *C. perfringens* (3 × 10^10^ cfu/mL) for five consecutive days. The birds in groups C and D had milder or absence of these gross and microscopic changes in various organs. In this aspect, Ref [34] found that the *C. perfringens* infected birds did not show typical gross and histological changes after oral gavage of anti-clostridial *IgYs* @ 3 mL/bird. The basic mechanism of EYAs activity against enteric pathogens involves binding with specific pathogens, followed by immobilization and, ultimately, a decrease in their growth [10]. The use of anti-clostridial *IgYs* provides protective effects against experimental NE infection and the findings of studies [14,34,35] suggested that *C. perfringens* and other enteric pathogens can be targeted through passive immunization by using purified *IgY* in birds.

## 5. Conclusions

The outcome of the current study demonstrated that the passive immunization by anti-clostridial EYAs can be effective in acute NE. EYAs can be beneficial to overcome antimicrobial resistance (AMR) issues without producing harmful effects in animals. For the production of antibiotic-residues free poultry and their products, future studies would be needed for a safer production and a reliable application of EYAs e.g., combined use of different EYAs and/or other alternatives e.g., enzymes and probiotics to investigate their therapeutic and growth-promoting roles in poultry.

## Figures and Tables

**Table 1:** Design and infection of the experimental birds.

Groups	Treatment	Route	Dose	Duration
A	-	-	-	-
B	*C. perfringens* type A	Per os	1 × 10^8^ cfu/mL	On days 17, 18 and 19
C	*C. perfringens* type A	Per os	1 × 10^8^ cfu/mL	On days 17, 18 and 19
	EYA	Per os	1mL/bird	On days 21, 22, 23 and 24
D	*C. perfringens* type A	Per os	1 × 10^8^ cfu/mL	On days 17, 18 and 19
	EYA	I/M	1mL/bird	On days 22 and 24

**Table 2:** Scoring results of various clinical signs and behavioral symptoms in birds.

Week	Signs	Score Range	Groups			
			A	B	C	D
1	Activeness	0–4	4.0 ± 0.0a	4.0 ± 0.0a	4.0 ± 0.0a	3.5 ± 0.53b
	Fecal consistency	0–4	4.0 ± 0.0a	4.0 ± 0.0a	4.0 ± 0.0a	4.0 ± 0.0a
	Desire for feed	0–4	4.0 ± 0.0a	4.0 ± 0.0a	4.0 ± 0.0a	4.0 ± 0.0a
	Total		12.0 ± 0.0	12.0 ± 0.0	12.0 ± 0.0	11.50 ± 0.53
2	Activeness	0–4	4.0 ± 0.0a	4.0 ± 0.0a	4.0 ± 0.0a	4.0 ± 0.0a
	Fecal consistency	0–4	4.0 ± 0.0a	4.0 ± 0.0a	3.70 ± 0.48b	4.0 ± 0.0a
	Desire for feed	0–4	4.0 ± 0.0a	4.0 ± 0.0a	4.0 ± 0.0a	4.0 ± 0.0a
	Total		12.0 ± 0.0	12.0 ± 0.0	11.70 ± 0.48	12.0 ± 0.0
3	Activeness	0–4	3.80 ± 0.42a	2.20 ± 0.42b	2.20 ± 0.42b	2.30 ± 0.48b
	Fecal consistency	0–4	4.0 ± 0.0a	2.10 ± 0.32b	2.20 ± 0.42b	2.20 ± 0.42b
	Desire for feed	0–4	3.90 ± 0.32a	2.30 ± 0.48b	2.40 ± 0.52b	2.20 ± 0.42b
	Total		11.70 ± 0.74	6.60 ± 1.22	6.80 ± 1.36	6.70 ± 1.32
4	Activeness	0–4	4.0 ± 0.0a	2.30 ± 0.48bc	2.70 ± 0.48b	2.20 ± 0.42c
	Fecal consistency	0–4	4.0 ± 0.0a	2.20 ± 0.42b	2.60 ± 0.52b	2.30 ± 0.48b
	Desire for feed	0–4	4.0 ± 0.0a	2.20 ± 0.42c	2.40 ± 0.52bc	2.70 ± 0.48b
	Total		12.0 ± 0.0	6.30 ± 0.74	7.70 ± 1.52	7.20 ± 1.38
5	Activeness	0–4	3.90 ± 0.32a	2.10 ± 0.32c	2.80 ± 0.42b	2.70 ± 0.48b
	Fecal consistency	0–4	3.80 ± 0.42a	2.00 ± 0.0c	2.70 ± 0.48b	2.30 ± 0.48bc
	Desire for feed	0–4	3.90 ± 0.32a	2.20 ± 0.42c	2.90 ± 0.32b	2.60 ± 0.52bc
	Total		11.60 ± 1.06	6.30 ± 0.74	8.40 ± 1.22	7.60 ± 1.48
**Grand Total**			**59.30 ± 1.80**	**43.60 ± 3.28**	**46.60 ± 4.58**	**45.0 ± 4.71**

Mean values with different letters showed significant difference (*p* ≤ 0.05).

**Table 3:** Mortality (%) in various groups of experiment.

Group	Number of Dead Birds					Total	Mortality (%)
	Week 1	Week 2	Week 3	Week 4	Week 5		
A	0.0 ± 0.0a	0.0 ± 0.0a	0.0 ± 0.0a	0.0 ± 0.0a	0.0 ± 0.0a	0	0
B	0.0 ± 0.0a	0.0 ± 0.0a	0.3 ± 0.5a	0.2 ± 0.4a	0.1 ± 0.3a	6	30
C	0.0 ± 0.0a	0.0 ± 0.0a	0.2 ± 0.4a	0.1 ± 0.3a	0.0 ± 0.0a	3	15
D	0.0 ± 0.0a	0.0 ± 0.0a	0.2 ± 0.4a	0.1 ± 0.3a	0.1 ± 0.3a	4	20

Mean values with different letters have significant difference (*p* ≤ 0.05).

**Table 4:** Relative weight of various organs (g) in the first sacrifice (Mean ± SD).

Group	Carcass Weight	Liver	Kidneys	Jejunum
A	618.60 ± 1.96a	4.08 ± 0.06d	0.91 ± 0.03d	8.89 ± 0.0d
B	467.10 ± 1.72d	4.82 ± 0.07a	1.05 ± 0.02a	10.54 ± 0.09a
C	530.80 ± 2.15b	4.27 ± 0.04c	0.96 ± 0.02c	9.45 ± 0.04c
D	507.10 ± 1.73c	4.47 ± 0.04b	1.00 ± 0.02b	9.84 ± 0.05b

Mean values with different letters have significant difference (*p* ≤ 0.05).

**Table 5:** Relative weight of various organs (g) in 2nd killing (Mean ± SD).

Group	Carcass Weight	Liver	Kidneys	Jejunum
A	1169.00 ± 4.99a	2.56 ± 0.03c	0.90 ± 0.01b	7.41 ± 0.05d
B	757.80 ± 6.17d	3.31 ± 0.06a	1.03 ± 0.03a	8.42 ± 0.06a
C	1019.20 ± 4.80b	2.60 ± 0.02c	0.92 ± 0.01b	7.83 ± 0.03c
D	969.90 ± 4.97c	2.70 ± 0.01b	0.92 ± 0.01b	7.91 ± 0.04b

Mean values with different letters have significant difference (*p* ≤ 0.05).

**Table 6:** Lesion scoring for the gross examination of various organs in birds.

Group	Liver		Kidney		Jejunum	
	CN	EN	CN	EN	HM	MD
A	−	−	−	−	−	−
B	+++	+++	+++	+++	+++	+++
C	+	++	+	++	++	++
D	++	++	++	++	++	++

**Scoring:** absence of lesion (0%) = −, mild (≤10%) = +, moderate (10–20%) = ++, severe (20–30%) = +++, CN = congestion; EN = enlargement; HM = hemorrhages; MD = mucosal damage.

## Data Availability

No new data were created or analyzed in this study. Data sharing is not applicable to this article.
